# Perioperative coagulation profiling and postoperative lower-extremity venous thrombosis risk prediction in glioma patients

**DOI:** 10.7150/jca.136435

**Published:** 2026-08-24

**Authors:** Ping Pu, Miao Yang, Hong Zhou, Yan Xiang, Shuai Wang, Shengqing Lv, Wei Yang

**Affiliations:** 1 Department of Neurosurgery, Xinqiao Hospital, Army Medical University, Chongqing, 400037, China.; 2 Department of Breast and Thyroid Surgery, Daping Hospital, Army Medical University, Chongqing, 400042, China.; 3 Institute of Pathology and Southwest Cancer Center, Southwest Hospital, Army Medical University, Chongqing, 400038, China.

**Keywords:** glioma, venous thrombosis, coagulation, IDH1, surgery

## Abstract

**Background:**

Lower-extremity venous thrombosis (LEVT) is highly prevalent among glioblastoma patients, creating a therapeutic dilemma in clinical management.

**Methods:**

We retrospectively analyzed preoperative coagulation parameters in primary glioma, trigeminal neuralgia, and meningioma patients. Glioma cohorts were stratified by IDH1 and LEVT status, with coagulation factor expression analyzed using public databases. Perioperative coagulation dynamics were evaluated, and an LEVT prediction model was constructed.

**Results:**

Glioma patients exhibited a hypercoagulable state compared with non-neoplastic and benign tumor controls, with IDH1-Wt cases showing particularly elevated fibrinogen levels versus IDH1-Mutant gliomas. Perioperative analysis revealed universal decreases in APTT and fibrinogen, while PT prolongation was exclusive to IDH1-Wt cases and APTT shortening predominated in IDH1-Mutant patients. Spatial and single-cell analyses demonstrated IDH1 mutation-specific overexpression patterns of coagulation factors with prognostic significance. LEVT patients were characterized by shorter preoperative APTT, lower postoperative fibrinogen, and distinct coagulation parameter trajectories. These findings informed the development of a high-performance predictive model incorporating perioperative coagulation markers for LEVT risk assessment.

**Conclusion:**

We characterized perioperative coagulation profiles in glioma patients and developed an accurate LEVT prediction model, enabling early intervention to improve outcomes.

## Introduction

Patients with malignancies frequently develop cancer-associated hypercoagulability 1_,_ characterized by persistent coagulation activation through tumor-host interactions that promote venous thromboembolism (VTE) 2. Lower extremity venous thrombosis (LEVT) constitutes approximately 90% of VTE cases, carrying a significant risk of pulmonary embolism 3. Since Trousseau's 1865 report, VTE has been recognized as the second leading cause of cancer-related mortality after disease progression 4. Tumor-mediated platelet activation, thrombin generation, and fibrin deposition combine with treatment effects and immobilization to fulfill Virchow's triad 5. Notably, glioma, particularly glioblastoma, carries the highest VTE risks among solid tumors 6.

As the most common primary CNS malignancy, high-grade gliomas exhibit aggressive behavior and poor prognosis 7. Although treatment advances have improved survival 8, complications such as LEVT, have become increasingly important 9, 10. Glioma patients have 2-3 times higher VTE risk than patients with other solid tumors and > 10-fold greater risk than the general population 11, 12, with 20-30% developing VTE during treatment, especially postoperatively or during chemo/radiotherapy 13. LEVT prolongs hospitalization, may require treatment modifications due to anticoagulation risks, and compromises outcome 14. Therefore, a thorough investigation of the pathological mechanisms, risk factors, prevention, and treatment strategies for thrombosis in glioma patients is urgently needed to improve clinical outcomes.

Glioma-associated thrombosis arises from complex tumor-host-treatment interactions, including tumor-secreted procoagulants (tissue factor, microparticles, cytokines) 15-17, patient comorbidities (hypertension, hyperlipidemia, diabetes) 6,9, and treatment factors (prolonged surgery, immobility, steroids/chemotherapy) 18, 19. Current VTE risk models (Khorana, Caprini RAM, IMPROVE, Padua Prediction scores) perform poorly in glioma patients due to inadequate tumor-specific considerations 20, 21. LEVT is typically diagnosed occurs post-symptom onset via D-dimer/ultrasound, whereas anticoagulation therapy carries significant hemorrhage risks 22. Given that 75% of LEVT cases occurring within three months post-diagnosis and that current models' limited predictive value (PPV < 35%), developing glioma-specific early prediction tools is critical for improving clinical outcomes 14, 23.

This study comprehensively characterizes perioperative coagulation profiles in glioma patients through: systematic comparisons with non-neoplastic and benign intracranial conditions; analysis of IDH1 subgroup variations and pre-to-postoperative dynamic changes; and evaluation of LEVT-associated coagulation patterns. We further investigate spatial distributions and single-cell expression of coagulation factors in glioma tissue. Finally, we develop and validate a predictive model integrating perioperative coagulation parameters for early postoperative LEVT risk assessment. These findings reveal intrinsic heterogeneity in glioma-associated coagulation dysfunction and provide a clinically actionable tool for LEVT prediction and prevention.

## Methods

### Patient recruitment

We retrospectively enrolled hospitalized patients from the Department of Neurosurgery at Xinqiao Hospital between June 2022 and December 2023. Patients with primary gliomas and meningiomas were included based on postoperative pathological diagnoses, while recurrent cases and malignant meningiomas (WHO grades II and III) were excluded. Trigeminal neuralgia patients were confirmed through typical symptoms and imaging to rule out secondary causes (surgical history was not considered). After applying coagulation-related inclusion/exclusion criteria to 176 glioma patients, 145 cases were analyzed for postoperative LEVT using vascular ultrasound. Coagulation parameters within three days after surgery were collected for 111 matched glioma patients who underwent postoperative coagulation testing, including Activated Partial Thromboplastin Time (APTT), Prothrombin Time (PT), Prothrombin Activity (PTA), International Normalized Ratio (INR), Fibrinogen (FIB), and Thrombin Time (TT) .Using the same inclusion and exclusion criteria, we selected 193 cases of primary benign meningioma from 287 meningioma patients with preoperative coagulation tests, and 100 cases from 146 trigeminal neuralgia patients with preoperative coagulation tests as controls. The sample size was determined by the number of all available cases meeting inclusion criteria during the study period. No formal power calculation was performed, as this was a descriptive analysis without a priori hypotheses.

### Propensity score matching and coagulation parameter analysis across diagnoses

Due to differences in sex, age distribution, and comorbidities (hypertension, diabetes, hyperlipidemia, smoking/alcohol history) among primary gliomas, TN, and benign meningiomas, we performed rigorous PSM. For primary gliomas versus TN or benign meningiomas, we assigned binary values to sex, hypertension, diabetes, hyperlipidemia, and smoking/alcohol use history, with age as a continuous variable. Data from 145 primary gliomas and 100 TN patients or 193 benign meningioma patients were imported into SPSS. Categorical variables (sex, age, comorbidities) underwent baseline testing (χ² or t-tests) **(Tables 1 and 2)**. PSM was conducted with a caliper of 0.5 for propensity scores (PS), followed by recalibration to 0.2 × PS standard deviation. Matched pairs were re-evaluated using χ² or t-tests **(Tables 3 and 4)**. Coagulation data from matched pairs were assessed for normality (Kolmogorov-Smirnov test), with paired t-tests or Wilcoxon tests applied based on distribution.

### Pre- vs. postoperative coagulation differences in glioma by IDH1 status

Among 145 primary gliomas, IDH1 status was categorized as wild-type (IDH1-Wt, n = 75), mutant (IDH1-Mut, n = 69), and 1 undetermined. After excluding incomplete pre/postoperative data, 111 patients (IDH1-Wt = 60, IDH1-Mut = 50, 1 undetermined) were analyzed. PSM was performed using age, sex, hypertension, diabetes, hyperlipidemia, and smoking/alcohol use history as covariates **(Tables 5 and 6)**. This yielded 49 matched preoperative and 39 post-operative pairs. Post-matching baseline differences were reassessed (χ²/t-tests) **(Tables 7 and 8)**. Coagulation parameters were compared using paired t-tests or Wilcoxon tests based on normality.

### Pre- vs. postoperative coagulation changes in gliomas

The 111 matched glioma patients (IDH1-Wt = 60, IDH1-Mut = 50, 1 undetermined) were evaluated for coagulation parameter changes. Normality tests (Kolmogorov-Smirnov test) guided the choice of paired t-tests or Wilcoxon tests for analysis.

### Coagulation factor expression in databases

Using the TCGA-LGG+GBM database (n = 667), we analyzed the expression of coagulation-related genes. Genes were ranked by expression and stratified by IDH1 status, then grouped by pathway (intrinsic, extrinsic, common). Differential expression between IDH1-Wt and IDH1-Mut groups was assessed. The Ivy-GAP database was used to examine regional expression patterns of F3, F8, F12, F5, and F13A1 across glioma subregions (https://glioblastoma.alleninstitute.org). Single-cell RNA sequencing data were downloaded from the publicly available GEO series GSE182109 (obtained from 18 glioma patients, including 2 LGG, 11 ndGBM, and 5 rGBM cases), and analyzed to determine cell-type-specific expression of highly expressed coagulation genes (F3, F8, F5, F13A1, F12, F10, F7). Finally, Kaplan-Meier survival analysis was performed in the TCGA dataset to evaluate the prognostic significance of elevated F8, F12, F3, and F13A1 expression in glioma patients (Gene-Protein, F3, Coagulation Factor III or Tissue Factor; F5, Coagulation Factor V; F7, Coagulation Factor VII; F8, Coagulation Factor VIII; F10, Coagulation Factor X; F12, Coagulation Factor XII; F13A1, Coagulation Factor XIII A subunit).

### Differences in perioperative coagulation between glioma patients with and without postoperative LEVT

This study analyzed coagulation parameters in 145 primary glioma patients with preoperative data (43 LEVT cases vs. 102 controls) and 111 patients with postoperative tests within 3 days (37 LEVT cases vs. 74 controls). Coagulation differences between groups were assessed using paired t-tests or Wilcoxon tests based on normality results from Kolmogorov-Smirnov testing. The analysis also examined pre-to-postoperative coagulation changes in 33 LEVT patients compared with 74 controls using identical statistical methods. To minimize bias, the diagnosis of LEVT was independently confirmed by two clinical ultrasound physicians who were blinded to the objectives of this study (blinded assessment).

### Risk factor analysis and predictive modeling for postoperative LEVT

For LEVT risk assessment, we evaluated multiple factors across 145 glioma patients, including demographic characteristics (age, sex), preoperative coagulation markers (APTT, PT, PTA, INR, TT, FIB), surgical parameters (operation duration, blood loss), postoperative status (limb muscle weakness), and pathological findings (IDH1 mutation status). Variables were numerically coded: sex (male = 1, female = 0), operation duration (≥ 290 minutes = 1, < 290 minutes = 0, median 290 minutes), blood loss (≥ 350mL = 1, < 350mL = 0, median 350ml), limb weakness (present = 1, absent = 0), and IDH1 status (wild-type = 1, mutant = 0). After excluding 4 incomplete cases (3 cases with limb weakness and 1 case missing IDH1 data), the final cohort comprised 141 patients. We conducted multicollinearity analysis, which led to excluding PTA and INR due to VIF > 5. Subsequent univariate logistic regression identified significant factors (p < 0.2) for inclusion in multivariate modeling. Predictive performance was evaluated through ROC analysis comparing two models: Model A incorporated preoperative coagulation parameters (APTT, TT, PT) along with clinical factors (age, limb weakness, operation duration); Model B used only clinical factors; and Model C used only preoperative coagulation parameters. This analytical approach was replicated in a subgroup of 104 patients with complete postoperative coagulation data (from original 111 cases), comparing Model A (postoperative TT plus clinical factors) against Model B (clinical factors alone) and Model C (postoperative TT alone) to evaluate the contribution of postoperative coagulation measurements. To evaluate the incremental predictive value of coagulation parameters, we statistically compared the areas under the ROC curves (AUC) between nested models using DeLong's nonparametric approach for correlated ROC curves, with all analyses performed in SPSS. Because our multivariate regression analysis involved 6 and 4 indicators respectively, the corresponding cohort of 141 and 104 patients met the sample size requirements.

### Statistical analysis

All normally distributed data are presented as mean ± standard deviation. Differences between groups with normal distribution were analyzed using t-tests (paired or unpaired). Non-normally distributed data are presented as median and interquartile range. Differences between paired non-normally distributed groups were assessed using the Wilcoxon matched-pairs signed-rank test, while differences between unpaired non-normally distributed groups were evaluated using the Mann-Whitney test. A P-value < 0.05 was considered statistically significant.

### Ethics

Our study was approved by the Ethics Committee of the Second Affiliated Hospital of Army Medical University (No. 2025-Yandi-046-01), and all research procedures complied with the Declaration of Helsinki.

## Results

### Glioma patients exhibit a preoperative hypercoagulable state compared with trigeminal neuralgia and primary benign meningioma patients

**Figure 1:**
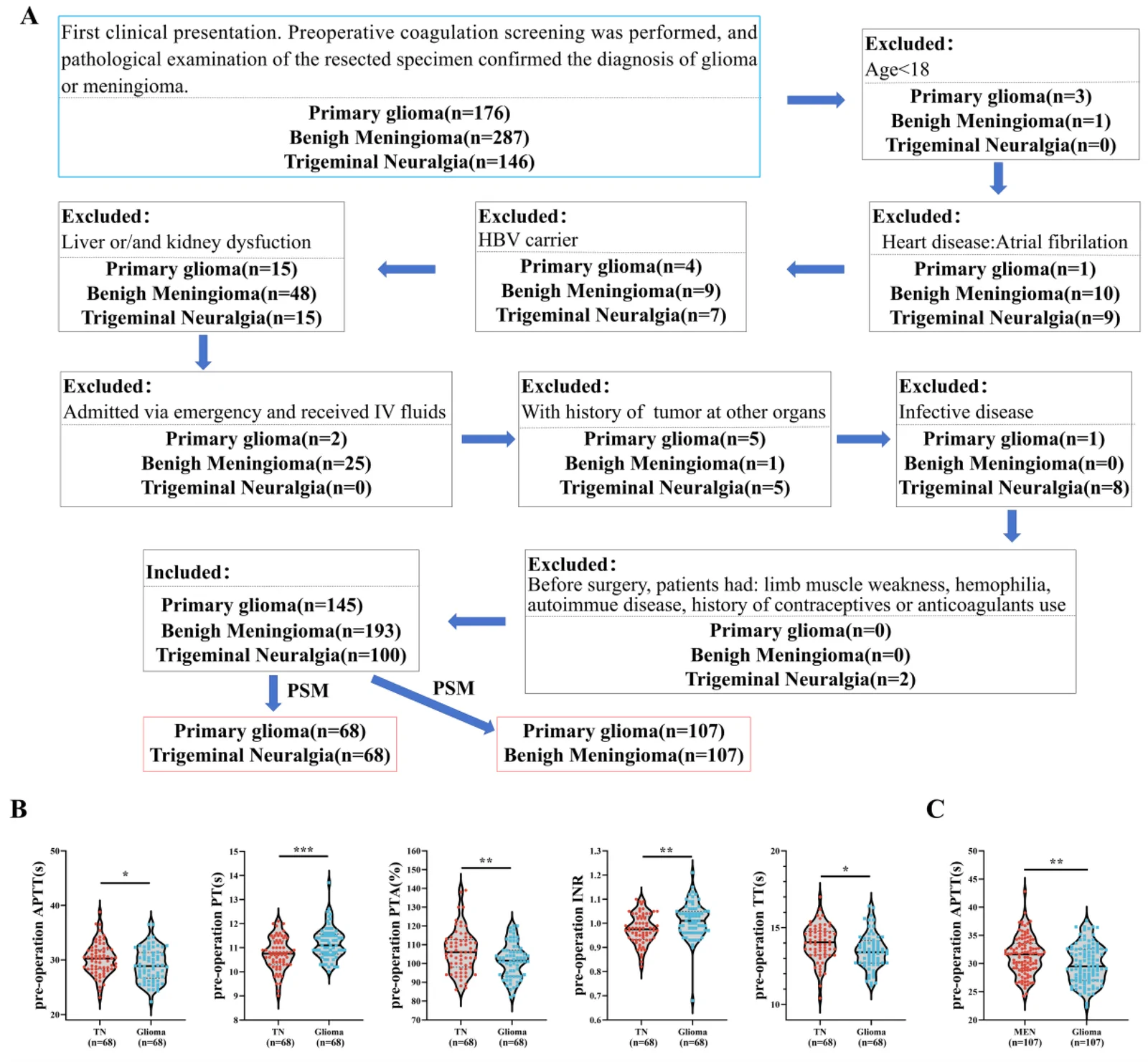
** Primary glioma patients exhibit a preoperative hypercoagulable state compared with trigeminal neuralgia and benign meningioma patients. A:** Consort flow diagram of the study on preoperative coagulation differences in patients with primary glioma, trigeminal neuralgia, and primary benign meningioma. **B:** Comparisons of preoperative coagulation parameters (APTT, PT, PTA, INR, and TT) between primary glioma patients and trigeminal neuralgia patients. **C:** Comparisons of preoperative APTT between primary glioma patients and benign meningioma patients, respectively. *, p < 0.05; **, p < 0.01; ***, p < 0.001.

From an initial cohort of 176 primary glioma, 287 benign meningioma, and 146 trigeminal neuralgia (TN) patients, 145 glioma, 193 meningioma, and 100 TN patients met the inclusion criteria. After propensity score matching (PSM), 68 TN-glioma and 107 meningioma-glioma pairs were analyzed (**Figure 1A**). Compared to TN patients, glioma patients showed significantly shorter TT (13.49 ± 1.16 vs. 14.00 ± 1.18, p = 0.012) and APTT (29.08 ± 3.06 vs. 30.18 ± 2.95, p = 0.050), reduced PTA (101.60 ± 9.37 vs. 106.40 ± 11.42, p = 0.010), prolonged PT (11.19 ± 0.67 vs. 10.74 ± 0.67, p = 0.0002), and elevated INR (1.013 ± 0.074 vs. 0.975 ± 0.062, p = 0.002), with no difference in FIB (**Figure 1B & S1A**). Relative to meningioma patients, glioma patients had shorter APTT (29.89 ± 3.42 vs. 31.38 ± 3.35, p = 0.002) but no significant differences in PT, PTA, INR, TT, or FIB (**Figure 1C & S1B**). These results suggest that glioma patients exhibit intrinsic coagulation pathway activation and prothrombin activity suppression compared to TN patients, whereas only intrinsic pathway activation is observed relative to meningioma patients.

### Differences in preoperative and postoperative coagulation function between IDH1-Mut and IDH1-Wt glioma patients

**Figure 2:**
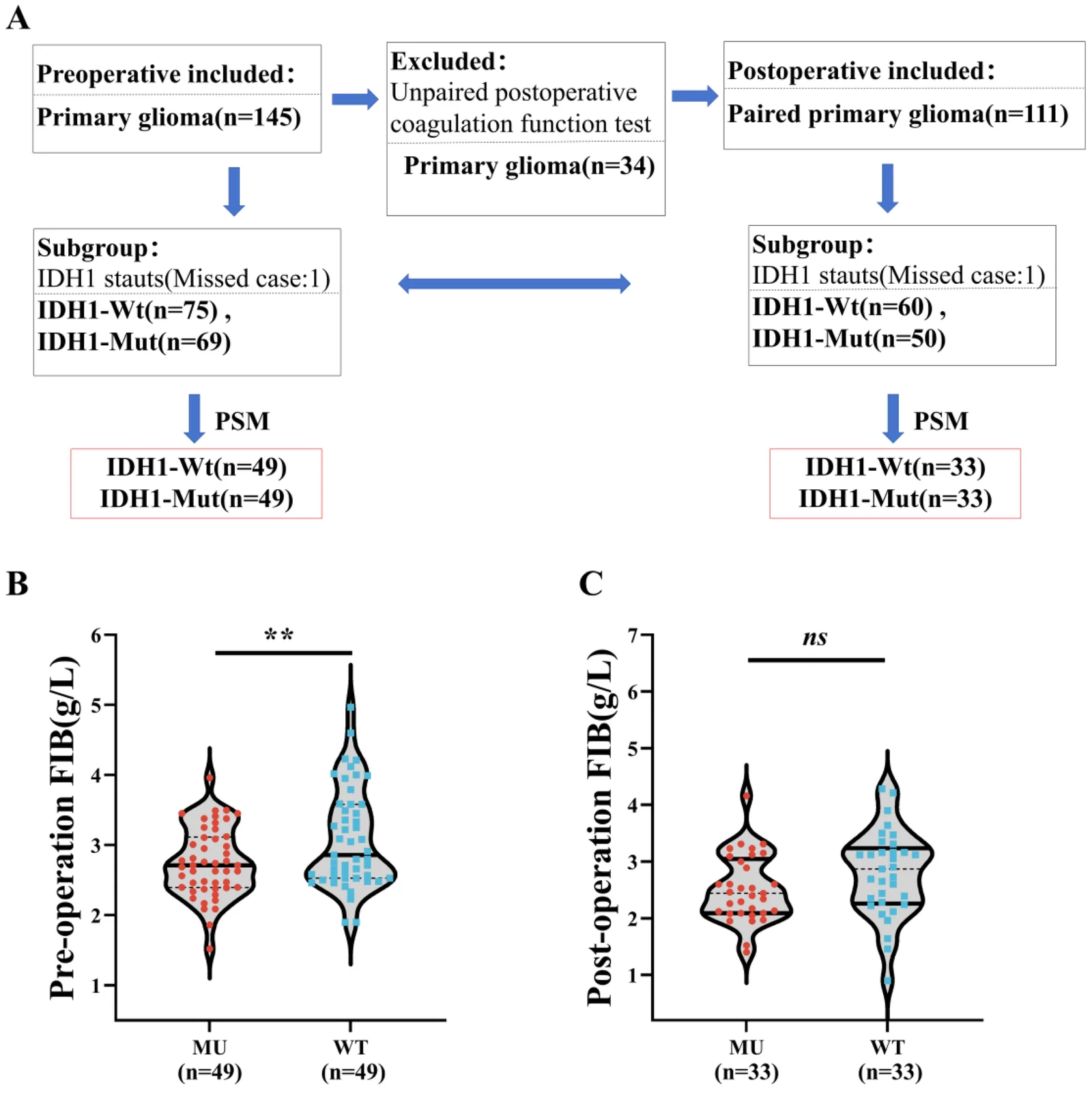
** Primary IDH1-Wt patients demonstrate a relatively hypercoagulable state compared to IDH1-Mut patients in the preoperative and postoperative periods. A:**Workflow of coagulation parameter analysis in glioma patients pre- and post-operation stratified by IDH1 mutation status (IDH1-Mut vs. IDH1-Wt). **B-C:** Comparisons of preoperative and postoperative FIB between primary IDH1-Mut and IDH1-Wt patients, respectively; **, p < 0.01.

Given the reported higher VTE risk in IDH1-Wt versus DH1-Mut gliomas 24, we compared their perioperative coagulation profiles. From 145 glioma patients, PSM yielded 49 matched pairs (**Figure 2A**). IDH1-Wt patients exhibited higher FIB levels (2.86 [2.53-3.58] vs. 2.71 [2.40-3.12], p = 0.009) but no differences in APTT, PT, PTA, INR, or TT (**Figure 2B&S2A**), suggesting a hypercoagulable state specific to the terminal coagulation pathway in IDH1-Wt tumors. Postoperative analysis of 110 patients (33 matched pairs after PSM) revealed no intergroup differences in coagulation parameters (**Figure 2C&S2B**), indicating surgical normalization of the preoperative FIB elevation.

### Perioperative changes of coagulation status in glioma patients

**Figure 3:**
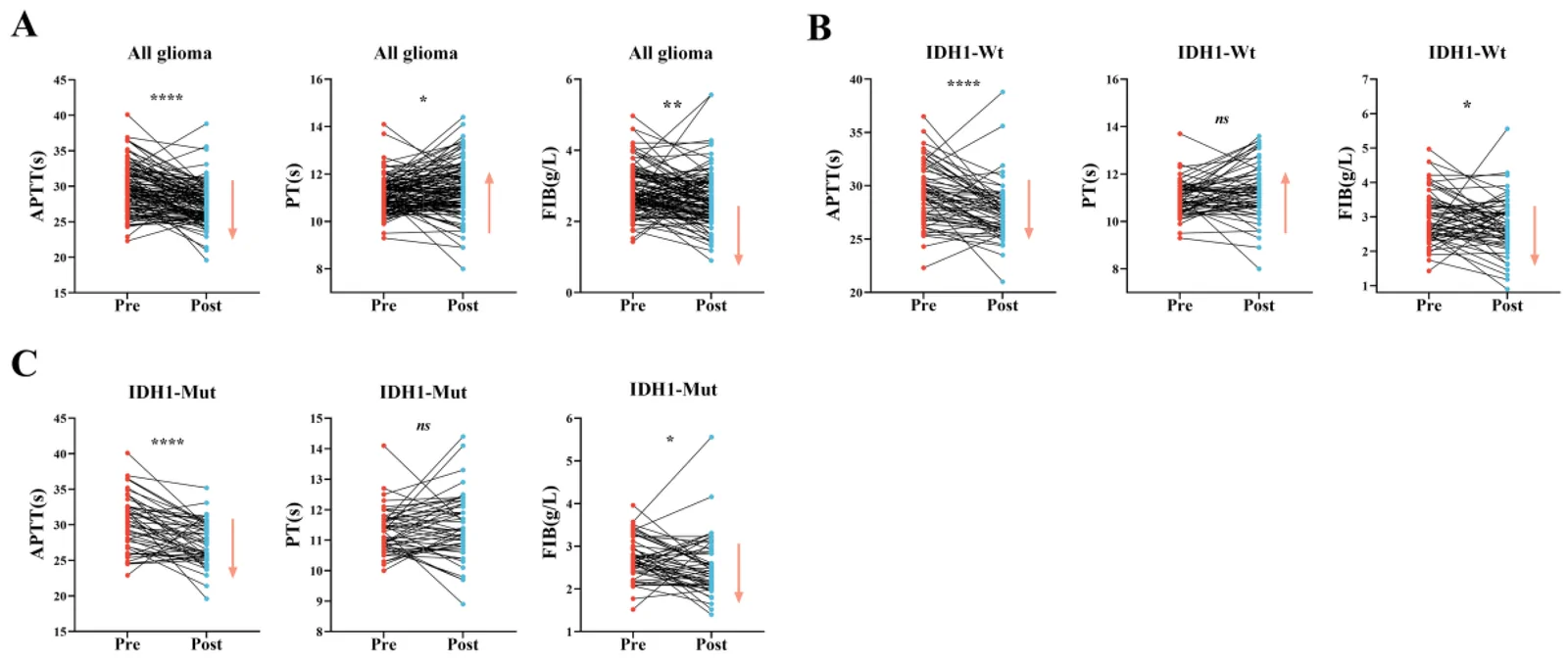
** Significant differences in coagulation status between preoperative and postoperative periods in primary glioma patients. A-C:** Comparisons of coagulation parameters (APTT, PT, and FIB) preoperative-postoperative changes in (A) all glioma patients (n = 111), (B) IDH1-Wt patients (n = 60), and (C) IDH1-Mut patients (n = 50); *, p < 0.05; **, p < 0.01; ****, p <0.0001.

Analysis of paired pre- and postoperative samples from 111 glioma patients revealed significant postoperative changes: decreased APTT (27.55 ± 2.82 vs. 29.79 ± 3.37, p < 0.0001) and FIB (2.65 [2.16-3.12] vs. 2.71 [2.46-3.29], p = 0.002), increased PT (11.20 [10.70-12.00] vs. 11.10 [10.70-11.50], p = 0.026) and INR (1.030 [0.970-1.110] vs. 1.011 [0.960-1.050], p = 0.006), and reduced PTA (97.0 [88.0-106.0] vs. 101.0 [95.0-108.0], p = 0.005), whereas TT remained stable (**Figure 3A&S3A**). Subgroup analysis showed these changes were more pronounced in IDH1-Wt patients (n = 60, **Figure 3B&S3B**), suggesting surgery-induced intrinsic pathway activation coupled with extrinsic pathway suppression. In contrast, IDH1-Mut patients (n = 50) exhibited only reduced FIB (2.42 [2.09-2.90] vs. 2.68 [2.40-3.04], p = 0.027) and APTT (27.46 ± 3.05 vs. 30.35 ± 3.91, p < 0.0001), indicating selective intrinsic pathway activation without extrinsic pathway involvement (**Figure 3C&S3C**). These findings demonstrate molecular subtype-specific coagulation activation mechanisms: IDH1-Wt gliomas exhibit combined intrinsic activation and extrinsic suppression, whereas IDH1-Mut cases show isolated intrinsic pathway activation following surgery.

### Expression patterns of coagulation factors in glioma tissue and cells

**Figure 4:**
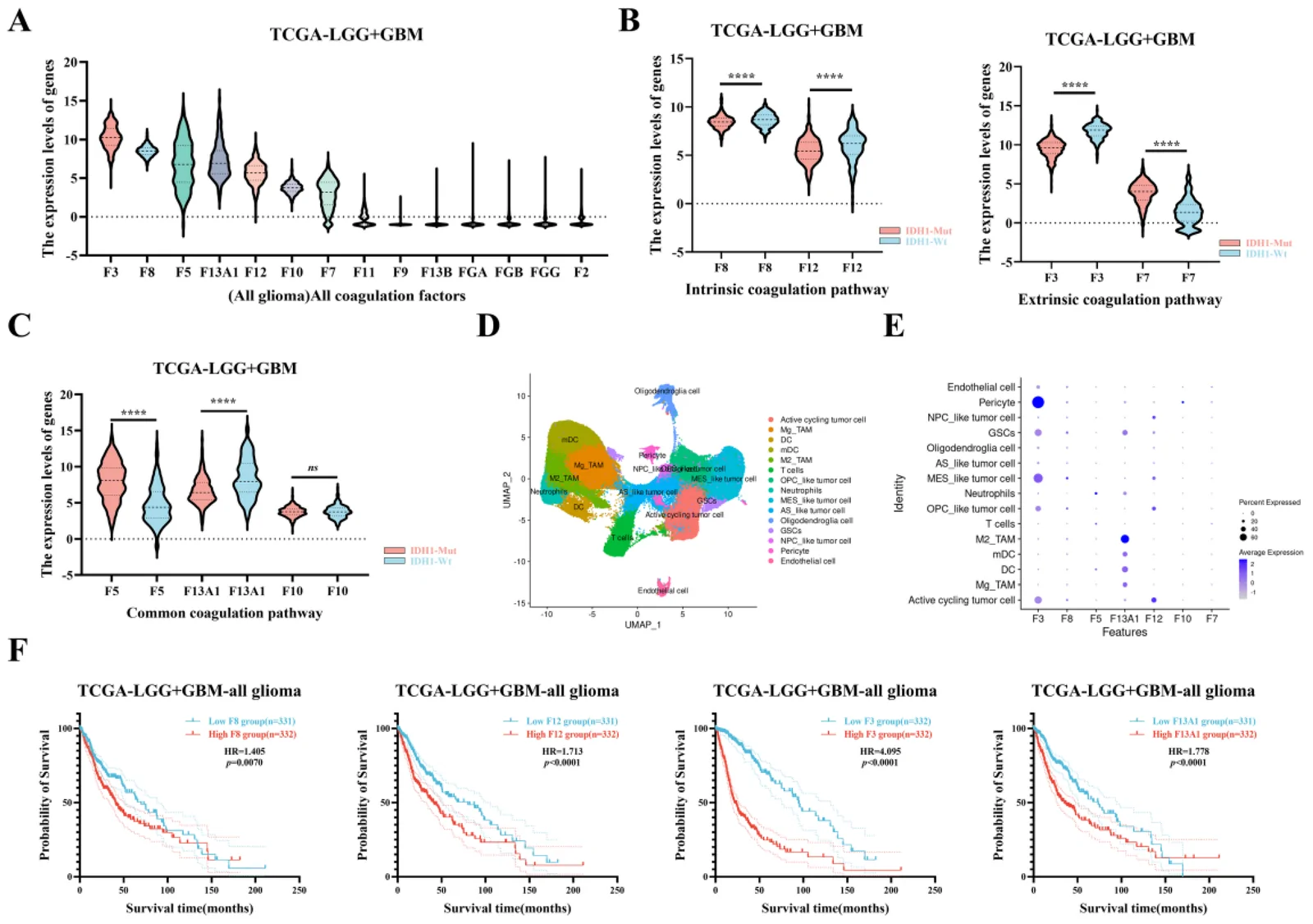
** Expression patterns of coagulation factors in glioma tissue. A:** Gene expression analysis of coagulation factors in all glioma patients. **B-C:** Comparative analysis of coagulation factor expression in intrinsic, extrinsic, and common pathways between IDH1-Mut and IDH1-Wt gliomas. **D-E:** Analysis of differentially expressed coagulation factor genes (F3, F8, F5, F13A1, F12, F10, F7) across cell subtypes using single-cell RNA sequencing databases. **F:** Association between highly expressed coagulation factors (F8, F12, F3, F13A1) and glioma patient prognosis. ****, p <0.0001.

Beyond tissue factor (F3) and calcium ions, malignant tumors can synthesize coagulation factors independently of hepatic production. TCGA analysis revealed elevated coagulation gene expression in gliomas (F3 > F8 > F5 > F13A1 > F12 > F10 > F7) with distinct pathway patterns: extrinsic (F3 and F7 upregulation), intrinsic (F8 and F12 upregulation), and common pathways (F5, F13A1 and F10 upregulation) **(Figure 4A)**. DH1-Wt gliomas showed significantly higher F8 and F12 (intrinsic), increased F3 but decreased F7 (extrinsic), and reduced F5 with elevated F13A1 (common pathway) versus IDH1-Mut cases **(Figure 4B-C)**. Single-cell analysis identified cell-specific expression: F3 in pericytes/tumor cells, F8/F10 in tumor subtypes, F5 in immune cells, and F13A1 in M2-TAMs **(Figure 4D-E)**. Critically, coagulation factor upregulation correlated with poorer survival in gliomas **(Figure 4F)**. These findings collectively provide a comprehensive picture of the intricate relationship between glioma molecular subtypes, spatially organized coagulation factor expression, and their clinical consequences.

### Relationship between postoperative LEVT and coagulation function in glioma patients

**Figure 5:**
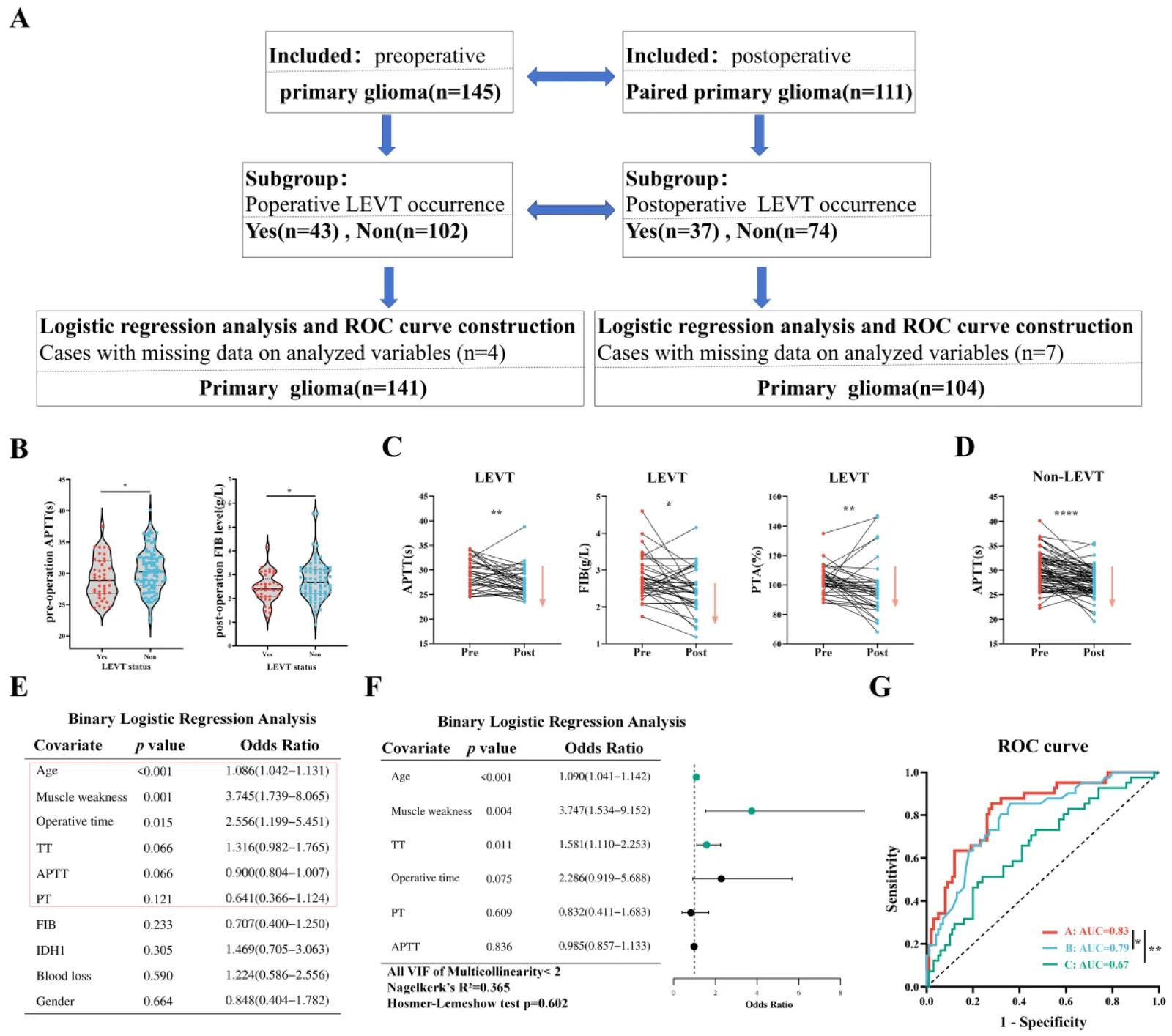
** Coagulation changes in glioma patients with vs. without postoperative LEVT and predictive model development for LEVT risk. A:** Workflow analysis of coagulation parameters dynamics in glioma patients with/without LEVT: Perioperative evaluation and temporal changes. **B:** Differences in preoperative APTT and postoperative FIB between glioma patients who developed postoperative LEVT and those who did not. **C:** Preoperative-to-postoperative changes in coagulation function (APTT, FIB, PTA) analyzed for glioma patients who developed postoperative LEVT.** D:** Preoperative-to-postoperative changes in APTT analyzed for glioma patients who did not develop postoperative LEVT. **E:** Univariate binary logistic regression analysis of preoperative coagulation factors influencing postoperative LEVT in glioma patients. **F:** Multivariate binary logistic regression analysis incorporating factors with p < 0.2 from univariate analysis. **G:** Comparison of LEVT prediction models for glioma patients: model A, incorporating preoperative coagulation parameters (TT, APTT, PT, age, postoperative muscle weakness, and operation duration), versus model B, without these parameters (age, postoperative muscle weakness, and operation duration), or model C, incorporating only preoperative coagulation parameters (TT, APTT, and PT). *, p < 0.05; **, p < 0.01; ****, p <0.0001.

Of 145 preoperative glioma patients, 43 (29.7%) developed LEVT, while 37 of 111 (33.3%) postoperative cases were LEVT-positive (**Figure 5A**). LEVT-positive patients showed significantly shorter preoperative APTT (29.20 ± 3.23 vs 30.45 ± 3.44, p = 0.044), and higher postoperative FIB (2.75±0.82 vs 2.44±0.61, p = 0.048) (**Figure 5B&S4A-B**) compared with LEVT-negative cases. Postoperative analysis revealed that LEVT patients exhibited greater coagulation changes: APTT reduction (27.55 ± 2.95 vs. 29.21 ± 3.06, p = 0.008), FIB decrease (2.40 [2.09-2.84] vs. 2.66 [2.46-3.08], p = 0.011), PTA decline (95.0 [85.5-102.0] vs. 103.0 [97.5-109.0], p = 0.004), and INR increase (1.040 [1.005-1.130] vs. 1.010 [0.960-1.040], p = 0.004) (**Figure 5C&S4C-D**). In contrast, LEVT-negative patients only showed APTT shortening (27.55 ± 2.78 vs. 30.08 ± 3.50, p < 0.001) (**Figure 5D&S4E**). These findings suggest postoperative extrinsic pathway activation specifically in LEVT patients.

### Construction of a predictive model for postoperative LEVT in glioma patients

We developed predictive models for postoperative LEVT in glioma patients using preoperative and postoperative parameters. Multivariate analysis identified advanced age, postoperative lower-limb weakness, and prolonged preoperative TT as independent risk factors (**Figure 5E-F**). The combined model (coagulation tests + clinical factors) showed superior discrimination (AUC 0.83, 95% CI: 0.75-0.90) versus clinical-only (AUC 0.79, p = 0.037) and coagulation-only (AUC 0.67, p = 0.001) models (**Figure 5G**). Postoperative analysis similarly revealed age, muscle weakness and prolonged TT as predictors (**Figure S5F-G**). While the combined model achieved AUC 0.82 (95% CI: 0.73-0.90), although its improvement over the clinical-only model (AUC 0.78, p = 0.267) was nonsignificant, it outperformed the TT-only model (AUC 0.63, p = 0.003) (**Figure S5H**).

## Discussion

Extensive research has established that malignant tumors induce a hypercoagulable state compared with healthy individuals 23. Our study expands this understanding through a rigorous retrospective analysis of a large patient cohort employing PSM to minimize confounding factors 25. By comparing glioma patients with matched controls with intracranial non-neoplastic lesions (trigeminal neuralgia) or benign tumors, we precisely characterized the unique preoperative coagulation profiles of gliomas 26, 27. Notably, while extracranial malignancies typically show hyperfibrinogenemia, gliomas demonstrate FIB levels comparable to those benign intracranial conditions but exhibit consistent APTT shortening, indicating intrinsic coagulation pathway activation as the primary mechanism of hypercoagulability.

Molecular stratification based on IDH1 status revealed important differences. IDH1-Wt glioblastomas, known for their aggressive clinical course with rapid recurrence and poor survival 9, 28, uniquely display elevated preoperative FIB levels, a finding absent in IDH1-Mut cases. This suggests upstream alterations in the coagulation cascade that amplify downstream to create a hypercoagulable state 17, 24, 29. Postoperatively, these differences disappeared, implying surgical attenuation of the tumor-mediated effects on coagulation 30. Given the hepatic origin of most coagulation factors, we speculate that a liver-glioma regulatory interplay may exist, in which different molecular subtypes may differentially influence hepatic function.

Our study revealed that glioma surgery may induce intrinsic coagulation pathway activation through multiple mechanisms, possibly including the release of procoagulants from resected tumor tissue, endothelial injury with collagen exposure, and hemostatic material application, which together may lead to FIB consumption via coagulation activation and secondary hyperfibrinolysis 30. Paradoxically, we observed extrinsic pathway suppression, which may be attributable to tumor removal (eliminating a major TF source), hemodilution, and perioperative vitamin K deficiency 31. Molecular stratification showed that IDH1-Mut patients exhibited only APTT shortening and FIB reduction postoperatively, whereas IDH1-Wt tumors demonstrated more pronounced extrinsic pathway changes consistent with their higher TF expression, highlighting how tumor biology interacts with surgical trauma to influence coagulation dynamics 16.

At the molecular level, gliomas show marked TF upregulation, particularly in IDH1-Wt tumors and perinecrotic mesenchymal-like cells 16, 32, 33. Single-cell analysis further revealed that TF is primarily expressed by various tumor cell populations, with the highest expression occurring in the most malignant MES-like cells that abundantly infiltrate necrotic areas 34. Surprisingly, among stromal cells, pericytes demonstrated even greater TF expression than the tumor cells themselves. Under physiological conditions, TF is mainly expressed by vascular adventitial cells (e.g., pericytes and fibroblasts) 35. In gliomas, pericytes routinely release TF-positive microparticles that activate the coagulation cascade while promoting vascular leakage and fibrin deposition 36. The frequent abnormal vascular proliferation and intravascular thrombosis observed in gliomas, particularly in IDH1-Wt subtypes, may result from TF release following tumor cell and pericyte apoptosis or detachment, thereby triggering thrombus formation 37. Although most coagulation factors are primarily liver-derived, our study identified elevated expression of multiple coagulation factors in glioma tissues. Notably, F8 and F12 of the intrinsic coagulation pathway were highly expressed in OPC-like and NPC-like cells at the invasive front, suggesting potential roles of intrinsic pathway molecules in glioma cell invasion. Conversely, the common pathway factors F5 and F13A1 were predominantly expressed in angiogenic regions, implying their functional importance in vascular remodeling or post-thrombotic dissolution processes 33, 34.

Glioma patients developing LEVT demonstrated distinct coagulation profiles: preoperative APTT shortening indicated pre-existing hypercoagulability, whereas postoperative TT prolongation and FIB depletion reflected thrombus formation and consumption. Importantly, LEVT patients uniquely showed extrinsic pathway suppression (decreased PTA and increased INR) alongside intrinsic pathway activation, suggesting that combined pathway dysregulation underlies thrombosis 17, 38. Multivariate analysis confirmed known risk factors (age and postoperative weakness) and newly identified TT prolongation as paradoxical LEVT predictors. This apparent contradiction likely represents a hypercoagulable-hyperfibrinolytic state, in which microthrombosis-generated FDPs interfere with thrombin activity while maintaining thrombin reserves for wound healing, which is consistent with the observed D-dimer elevation and prior reports of IDH1-Wt microthrombosis. Our coagulation parameter-based predictive model outperformed existing VTE risk tools (AUC 0.80) 21, providing superior preoperative LEVT risk stratification. This advance enables timely intervention to prevent pulmonary embolism, addressing the critical need for glioma management.

### Limitations

This study has several limitations. As this was a single-center retrospective study, our findings require validation through prospective multicenter studies. After PSM, the sample sizes of some subgroups were significantly reduced, necessitating future validation with larger cohorts. Additionally, our study focused solely on coagulation function analysis without investigating fibrinolytic markers, which warrants further exploration in future studies. Lastly, we classified patients into IDH1 wild-type and mutant groups solely by immunohistochemistry using the IDH1 R132H antibody, which will undoubtedly introduce classification bias. Therefore, future studies employing more precise IDH1 molecular subtyping would provide stronger support for our findings.

## Conclusion

Our study reveals distinct preoperative coagulation profiles in glioma patients compared with non-neoplastic/benign intracranial conditions, and identifies IDH1 mutation-specific perioperative coagulation patterns (preoperative to postoperative day 3) associated with LEVT development. Through multi-omics analysis, we characterized spatial/cell-type-specific coagulation factor expression patterns and their prognostic value. Most importantly, we developed and validated a clinically actionable predictive model incorporating perioperative coagulation parameters for early in-hospital LEVT detection, providing a timely intervention framework that addresses a critical unmet need in glioma management.

## Supplementary Material

Supplementary figures.

## Figures and Tables

**Table 1:** Raw baseline characteristics of patients with trigeminal neuralgia and primary glioma.

Characteristics	Trigeminal neuralgia glioma (n = 100)	Primary (n = 145)	p value
**Age(year)**	63.00±10.80	51.92±12.56	8.40e-12
**Sex**			
Men	44 (44.0)	86 (59.3)	
Women	56 (56.0)	59 (40.7)	0.018
**Hypertension**			
Yes	29 (29.0)	26 (17.9)	
No	71 (71.0)	119 (82.1)	0.041
**Diabetes mellitus**			
Yes	7 (7.0)	6 (4.1)	
No	93 (93.0)	139 (95.9)	0.326
**Hyperlipidemia**			
Yes	6 (6.0)	4 (2.8)	
No	94 (94.0)	141 (97.2)	0.208
**Tobacco and alcohol use**			
Yes	15 (15.0)	48 (33.1)	
No	85 (85.0)	97 (66.9)	1.44e-3

**Table 2:** Raw baseline characteristics of patients with Benign meningioma and primary glioma.

Characteristics	Benign meningioma (n = 193)	Primary glioma (n = 145)	p value
**Age (year)**	53.52±11.67	51.92±12.56	0.226
**Sex**			
Men	144 (74.6)	86 (59.3)	
Women	49 (25.4)	59 (40.7)	2.94e-10
**Hypertension**			
Yes	38 (19.7)	26 (17.9)	
No	155 (80.3)	119 (82.1)	0.683
**Diabetes mellitus**			
Yes	17 (8.8)	6 (4.1)	
No	176 (91.2)	139 (95.9)	0.092
**Hyperlipidemia**			
Yes	3 (1.6)	4 (2.8)	
No	190 (98.4)	141 (97.2)	0.701
**Tobacco and alcohol use**			
Yes	23 (11.9)	48 (33.1)	
No	170 (88.1)	97 (66.9)	2.00e-6

**Table 3:** Baseline characteristics of patients with trigeminal neuralgia and primary glioma after PSM.

Characteristics	Trigeminal neuralgia (n = 68)	Primary glioma (n = 68)	p value
**Age(year)**	59.15±9.36	59.47±9.91	0.845
**Sex**			
Men	38 (55.9)	35 (51.5)	
Women	30 (44.1)	33 (48.5)	0.606
**Hypertension**			
Yes	18 (26.5)	17 (25.0)	
No	50 (73.5)	51 (75.0)	0.844
**Diabetes mellitus**			
Yes	4 (5.9)	3 (4.4)	
No	64 (94.1)	65 (95.6)	1.000^a^
**Hyperlipidemia**			
Yes	2 (2.9)	3 (4.4)	
No	66 (97.1)	65 (95.6)	1.000^a^
**Tobacco and alcohol use**			
Yes	14 (20.6)	15 (22.1)	
No	54 (79.4)	53 (77.9)	0.834

^a^ Continuity correction for chi-square test.

**Table 4:** Baseline characteristics of patients with trigeminal neuralgia and primary glioma after PSM

Characteristics	Benign meningioma (n = 107)	Primary glioma (n = 107)	p value
**Age(year)**	51.02±11.75	52.29±12.19	0.438
**Sex**			
Men	58 (54.2)	58 (54.2)	
Women	49 (45.8)	49 (45.8)	1.000
**Hypertension**			
Yes	23 (21.5)	19 (17.8)	
No	84 (78.5)	88 (82.2)	0.491
**Diabetes mellitus**			
Yes	5 (4.7)	4 (3.7)	
No	102 (95.3)	103 (96.3)	1.000^a^
**Hyperlipidemia**			
Yes	1 (0.9)	0 (0.0)	
No	106 (99.1)	107 (100.0)	1.000^a^
**Tobacco and alcohol use**			
Yes	23 (21.5)	26 (24.3)	
No	84 (78.5)	81 (75.7)	0.625

^a^ Continuity correction for chi-square test.

**Table 5:** Raw baseline characteristics of preoperative glioma patients with IDH1-Wt and IDH1-Mut

Characteristics	IDH1-Wt (n = 75)	IDH1-Mut (n = 69)	p value
**Age(year)**	54.51±12.45	49.59±11.63	0.016
**Sex**			
Men	50 (66.7)	35 (50.7)	
Women	25 (33.3)	34 (49.3)	0.052
**Hypertension**			
Yes	17 (22.7)	9 (13.0)	
No	58 (77.3)	60 (87.0)	0.134
**Diabetes mellitus**			
Yes	4 (5.3)	2 (2.9)	
No	71 (94.7)	67 (97.1)	0.754^a^
**Hyperlipidemia**			
Yes	3 (4.0)	1 (1.4)	
No	72 (96.0)	68 (98.6)	0.672^a^
**Tobacco and alcohol use**			
Yes	25 (33.3)	17 (24.6)	
No	50 (66.7)	52 (74.4)	0.034

^a^ Continuity correction for chi-square test.

**Table 6:** Raw baseline characteristics of postoperative glioma patients with IDH1-Wt and IDH1-Mut

Characteristics	IDH1-Wt (n = 60)	IDH1-Mut (n = 50)	p value
Age (year)	55.53±11.63	49.02±11.49	0.004
Sex			
Men	39 (65.0)	23 (46.0)	
Women	21 (35.0)	27 (54.0)	0.045
Hypertension			
Yes	16 (26.7)	7 (14.0)	
No	44 (73.3)	43 (86.0)	0.104
Diabetes mellitus			
Yes	3 (5.0)	1 (2.0)	
No	57 (95.0)	49 (98.0)	0.745a
Hyperlipidemia			
Yes	3 (5.0)	1 (2.0)	
No	57 (95.0)	49 (98.0)	0.745a
Tobacco and alcohol use			
Yes	24 (40.0)	11 (22.0)	
No	36 (60.0)	39 (78.0)	0.044

^a^ Continuity correction for chi-square test.

**Table 7:** Baseline characteristics of preoperative glioma patients with IDH1-Wt and IDH1-Mut after PSM

Characteristics	IDH1-Wt (n = 49)	IDH1-Mut (n = 49)	p value
Age(year)	52.76±12.12	53.00±10.68	0.916
Sex			
Men	28 (57.1)	32 (65.3)	
Women	21 (42.9)	17 (34.7)	0.407
Hypertension			
Yes	8 (16.3)	17 (34.7)	
No	41 (83.7)	32 (65.3)	0.790
Diabetes mellitus			
Yes	4 (8.2)	2 (4.1)	
No	45 (91.8)	47 (95.9)	0.673a
Hyperlipidemia			
Yes	2 (4.1)	1 (2.0)	
No	47 (95.9)	48 (98.0)	1.000a
Tobacco and alcohol use			
Yes	18 (36.7)	17 (34.7)	
No	31 (63.3)	32 (65.3)	0.833

^a^ Continuity correction for chi-square test.

**Table 8:** Baseline characteristics of postoperative glioma patients with IDH1-Wt and IDH1-Mut after PSM

Characteristics	IDH1-Wt (n = 33)	IDH1-Mut (n = 33)	p value
**Age(year)**	52.42±10.45	54.12±10.07	0.504
**Sex**			
Men	22 (66.7)	20 (60.6)	
Women	11 (33.3)	13 (39.4)	0.609
**Hypertension**			
Yes	5 (15.2)	7 (21.2)	
No	28 (84.8)	26 (78.8)	0.523
**Diabetes mellitus**			
Yes	0 (0.0)	1 (3.0)	
No	33 (100.0)	32 (97.0)	1.000^a^
**Hyperlipidemia**			
Yes	3 (9.1)	1 (3.0)	
No	30 (90.9)	32 (97.0)	0.606^a^
**Tobacco and alcohol use**			
Yes	14 (42.4)	11 (33.3)	
No	19 (57.6)	22 (66.7)	0.447

^a^ Continuity correction for chi-square test
