# Supplementary material for: Perioperative coagulation profiling and postoperative lower-extremity venous thrombosis risk prediction in glioma patients
Source: J Cancer. 2026 Aug 24;17(9):1558–69. doi: 10.7150/jca.136435 (PMC13573301; doi:10.7150/jca.136435)

Figure S1. Non-differential preoperative coagulation parameters in primary glioma vs. trigeminal neuralgia/benign meningioma

A: Comparisons of preoperative FIB between primary glioma patients and trigeminal neuralgia patients. **B:** Comparisons of preoperative coagulation parameters (PT, PTA, INR, TT, and FIB) between primary glioma patients and benign meningioma patients, respectively.

Figure S2. No significant differences in pre-/postoperative coagulation between IDH1-Mut and IDH1-Wt gliomas

A-B: Comparisons of preoperative and postoperative coagulation parameters (APTT, PT, PTA, INR, and TT) between primary IDH1-Mut and IDH1-Wt patients, respectively.

Figure S3. Comparative analysis of preoperative vs postoperative coagulation profiles in primary glioma

A-C: Comparisons of coagulation parameters(PTA,INR and TT) preoperative-postoperative changes in (A) all glioma patients (n=111), (B) IDH1-Wt patients (n=60), and (C) IDH1-Mut patients (n=50); *, p<0.05; **, p<0.01; .

Figure S4. Perioperative coagulation differences in glioma patients with/without LEVT and predictive model construction

A-B: Differences in preoperative coagulation parameter(PT, PTA, INR, TT, and FIB) and postoperative coagulation parameter(APTT, PT, PTA, INR, and TT) between glioma patients who developed postoperative LEVT and those who did not. **C-D:** Preoperative-to-postoperative changes in coagulation parameter(PT, TT, and INR) analyzed for glioma patients who developed postoperative LEVT. **E:** Preoperative-to-postoperative changes in coagulation parameter(PT, PTA, INR, TT, and FIB) analyzed for glioma patients who did not develop postoperative LEVT. **F:** Univariate binary logistic regression analysis of postoperative coagulation factors influencing postoperative LEVT in glioma patients. **G:** Multivariate binary logistic regression analysis incorporating factors with p<0.2 from univariate analysis. **H:** Comparison of LEVT prediction models for glioma patients: model A incorporating postoperative coagulation parameters (TT, age, postoperative muscle weakness, and operation duration) versus model B without these parameters(age, postoperative muscle weakness, and operation duration) or model C only postperative TT. *，p<0.05, ** p<0.01.

Figure S1

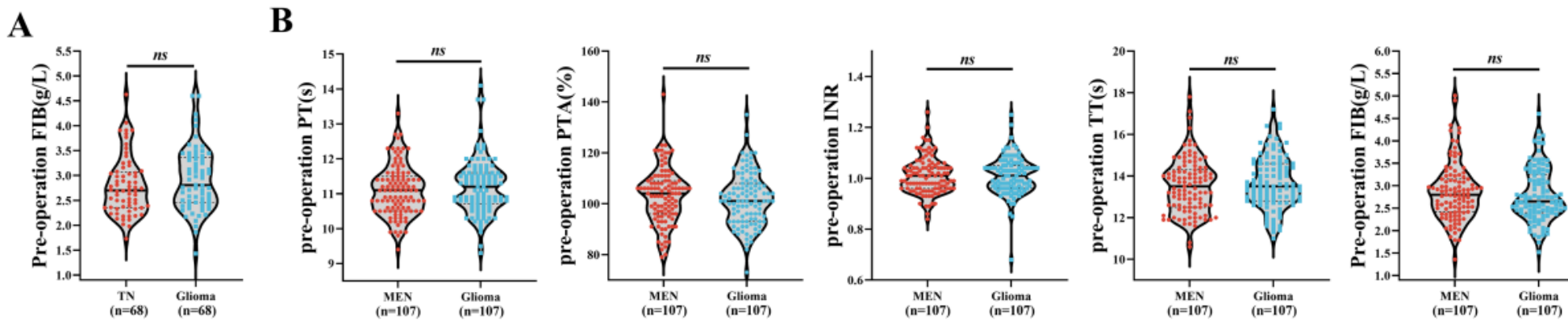

Figure S2

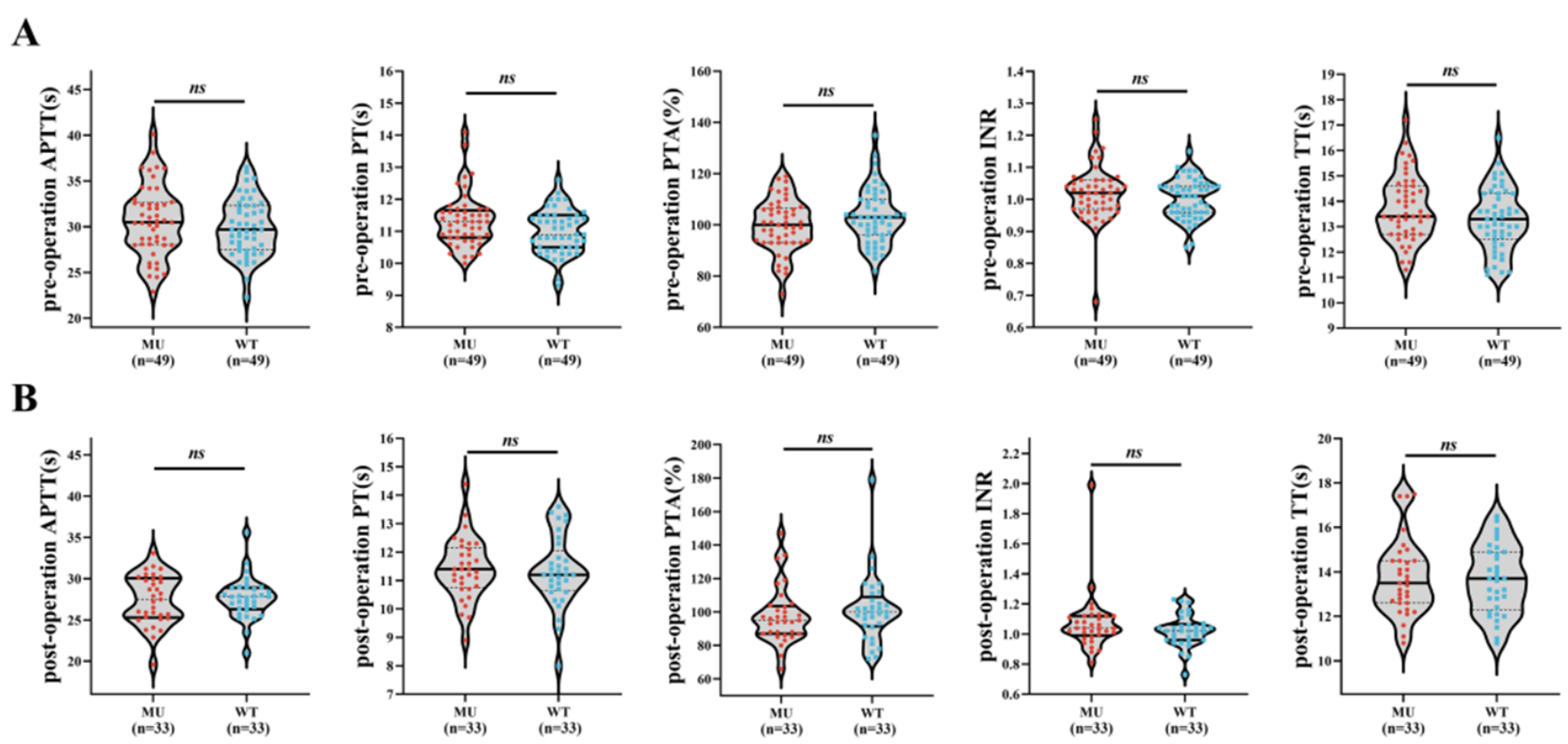

Figure S3

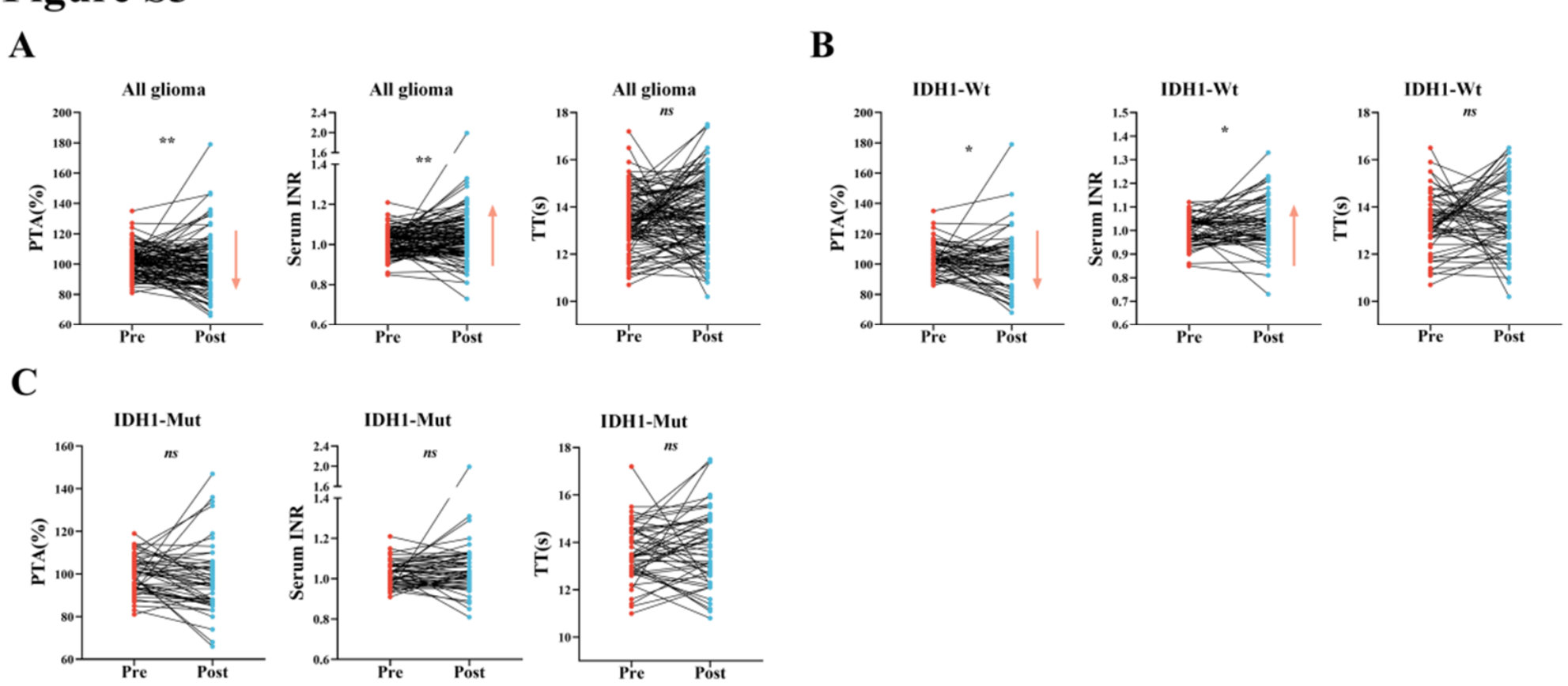

Figure S4

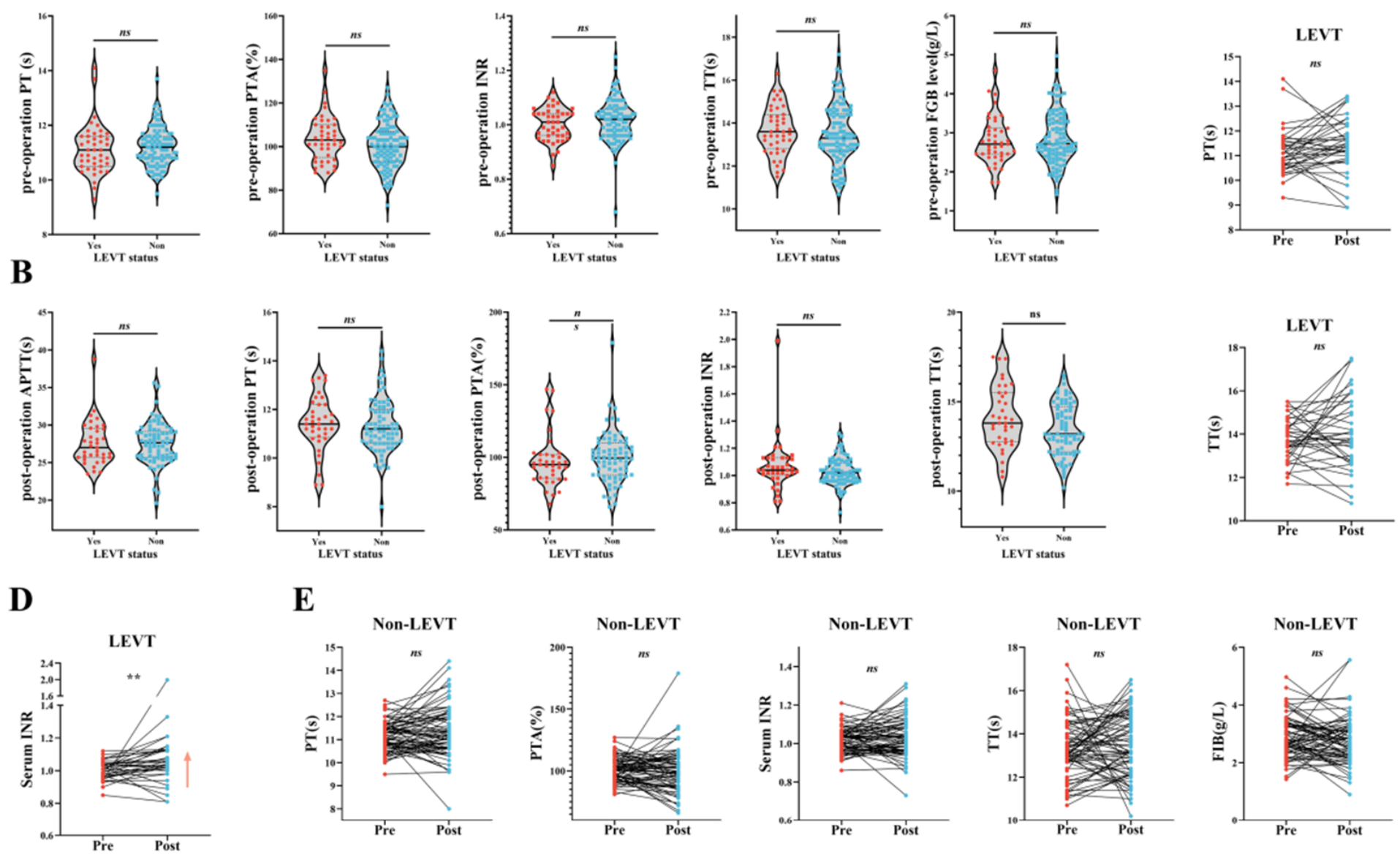

F

Binary Logistic Regression Analysis

Covariate	*p* value	Odds Ratio
Age	0.001	1.082(1.034−1.133)
Muscle weakness	0.001	4.348(1.761−10.753)
Operative time	0.011	3.071(1.292−7.304)
TT	0.018	1.404(1.061−1.859)
PT	0.576	1.118(0.756−1.652)
Blood loss	0.773	1.132(0.489−2.617)
Gender	0.800	0.898(0.390−2.067)
APTT	0.815	0.980(0.827−1.161)
IDH1	0.817	1.103(0.482−2.525)

G

Binary Logistic Regression Analysis

Covariate	*p* value	Odds Ratio
Age	0.001	1.084(1.032−1.138)
Muscle weakness	0.012	3.932(1.348−11.475)
TT	0.022	1.504(1.061−2.132)
Operative time	0.198	2.011(0.694−5.829)

All VIF of Multicollinearity< 2
Nagelkerk's R^2=0.374
Hosmer-Lemeshow test p=0.494

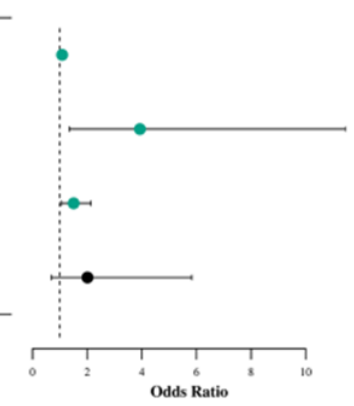

H

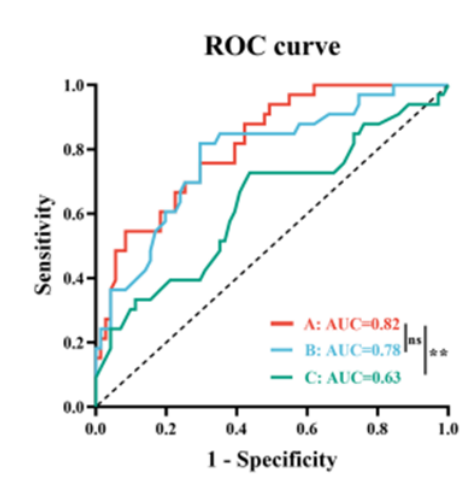

Supplement: Supplementary file 1 [file jcav17p1558s1.pdf]
